# Designing Electronic Structures of Multiscale Helical Converters for Tailored Ultrabroad Electromagnetic Absorption

**DOI:** 10.1007/s40820-024-01513-2

**Published:** 2024-09-26

**Authors:** Zhaobo Feng, Chongbo Liu, Xin Li, Guangsheng Luo, Naixin Zhai, Ruizhe Hu, Jing Lin, Jinbin Peng, Yuhui Peng, Renchao Che

**Affiliations:** 1 Key Laboratory of Jiangxi Province for Persistent Pollutants Control and Resources Recycle, School of Environmental and Chemical Engineering, Nanchang Hangkong University, Nanchang, 330063 People’s Republic of China; 2 School of Physics and Materials, Nanchang University, Nanchang, 330031 People’s Republic of China; 3 Key Laboratory of Nondestructive Testing, Ministry of Education, Nanchang Hangkong University, Nanchang, 330063 People’s Republic of China; 4 Laboratory of Advanced Materials, Shanghai Key Lab of Molecular Catalysis and Innovative Materials, Academy for Engineering and Technology, Fudan University, Shanghai, 200438 People’s Republic of China

**Keywords:** Metal–nonmetal co-doping, 3*d*–2*p* orbital coupling, Spin polarization, Helical structure, Broadband EM wave absorption

## Abstract

Atomic-scale doping strategies and structure design play pivotal roles in tailoring the electronic structure and physicochemical property of electromagnetic wave absorption (EMWA) materials. However, the relationship between configuration and electromagnetic (EM) loss mechanism has remained elusive. Herein, drawing inspiration from the DNA transcription process, we report the successful synthesis of novel in situ Mn/N co-doped helical carbon nanotubes with ultrabroad EMWA capability. Theoretical calculation and EM simulation confirm that the orbital coupling and spin polarization of the Mn–N_4_–C configuration, along with cross polarization generated by the helical structure, endow the helical converters with enhanced EM loss. As a result, HMC-8 demonstrates outstanding EMWA performance, achieving a minimum reflection loss of −63.13 dB at an ultralow thickness of 1.29 mm. Through precise tuning of the graphite domain size, HMC-7 achieves an effective absorption bandwidth (EAB) of 6.08 GHz at 2.02 mm thickness. Furthermore, constructing macroscale gradient metamaterials enables an ultrabroadband EAB of 12.16 GHz at a thickness of only 5.00 mm, with the maximum radar cross section reduction value reaching 36.4 dB m^2^. This innovative approach not only advances the understanding of metal–nonmetal co-doping but also realizes broadband EMWA, thus contributing to the development of EMWA mechanisms and applications.

**Supplementary Information:**

The online version contains supplementary material available at 10.1007/s40820-024-01513-2.

## Highlights

- The energy conversion mechanism is thoroughly analyzed, with a detailed quantitative characterization of the dissipation capacities of polarization, conduction, and magnetic loss.
- Inspired by DNA transcription, atom and geometry configurations co-modulating multi-scale helical converters achieve the *RL*_*min*_ of −63.13 dB at 1.29 mm, and the maximum RCS reduction value reach 36.4 dB m^2^.
- Orbital coupling, spin and cross polarization synergize to realize a 6.08 GHz EAB, further expanding to ultrabroad electromagnetic wave absorption of 12.16 GHz through metamaterial design*.*

**Supplementary Information:**

The online version contains supplementary material available at 10.1007/s40820-024-01513-2.

## Graphical abstract

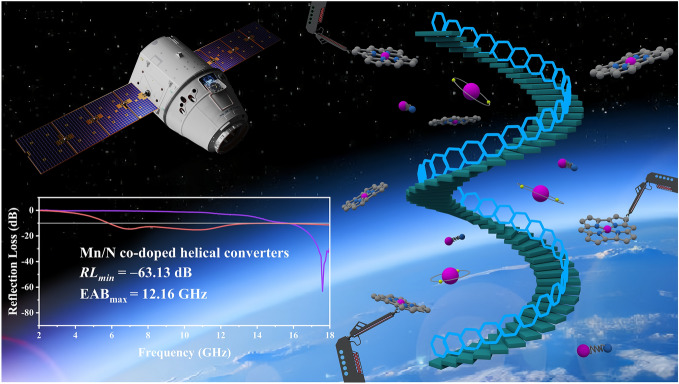

## Introduction

The advent of 5G technology, coupled with the proliferation of wireless communication technology and high-power electronic devices, including those enabling the Internet of Things, has led to a surge in electromagnetic (EM) pollution [1–9]. Despite some advancements in EM simulation and understanding loss mechanisms, the practical application of electromagnetic wave absorbing (EMWA) materials remains challenging.

Atomic doping strategies offer a promising avenue for enhancing polarization loss and EMWA performance [10, 11]. This approach induces lattice distortion, strain, and active point defects, leading to the creation of numerous dipole centers and in-plane topological defects. These mechanisms accelerate electron accumulation, trigger Maxwell polarization, and ultimately modulate the EM properties of materials [12]. Notably, metal–nitrogen–carbon (M–N–C) configurations have garnered significant attention across various fields owing to their high surface free energy, ultrahigh atom utilization, and the quantum size effect [13–15]. For instance, Shi et al. demonstrated the efficacy of an axial La–N–Cl/GNC structure boasting an ultralightweight property (0.084 g cm^-3^) and a broad effective absorption bandwidth (EAB*,* *RL* ≤  −10 dB) of 6.16 GHz at 2.36 mm [16]. Manganese is particularly renowned for its abundant valence electrons, which facilitate electron delocalization and the formation of high-spin states. The presence of additional unpaired electrons in manganese ions significantly increases the spin magnetic moment and electromagnetic wave (EMW) attenuation [17]. Consequently, Mn–N_4_–C species exhibit rich orbital couplings and spin interactions, holding promise for high-performance EMWA material development [18, 19]. The orbital coupling between the 3*d* orbitals of Mn and the 2*p* orbitals of N induces alterations in charge arrangement, generating novel electronic states (*d*-bands) near the Fermi level. This significantly enhances conductivity and dielectric loss [20]. Additionally, electronic spin, an intrinsic property of electrons, emerges as a promising avenue for modulating EMWA performance. Spin polarization, characterized by distinct asymmetric behavior in different spin directions, fosters the formation of high-spin states and augments EM attenuation [21–23]. Strategic atomic configuration design facilitates EM synergy, bolstering EMWA performance.

Beyond intrinsic atomic configurations, the geometric configuration of absorbers plays a pivotal role in EMWA performance. Helical materials, characterized by unique symmetry and EM coupling property, have garnered research attention. Helical structures contribute to additional EM loss by simultaneously inducing electric and magnetic polarization [24, 25]. Notably, Zuo et al. constructed the materials with helical nanotubes and investigated the impact of spatial configuration on permittivity, revealing additional cross polarization and a broad EAB of 6.7 GHz [26]. The results indicated that the helical configuration effectively diminishes the frequency sensitivity of EMWA materials, facilitating broadband absorption. Moreover, Huang et al. demonstrated that the EM field exhibited a uniform distribution along the length direction within tubular structures, as evidenced by EM simulations. However, the introduction of a helical structure disrupts this original EM field distribution, leading to its redistribution along the direction of the helical threads [27]. This phenomenon induces a robust EM resonance, further enhancing EMW attenuation. In essence, the helical structure not only facilitates the reconfiguration of the EM field distribution but also triggers strong EMW attenuation.

In this study, drawing inspiration from the DNA transcription process, we have developed unique helical converters by ingeniously manipulating both atomic and geometrical configurations. Specifically, the incorporation of Mn–N_4_–C species altered the electron distribution locally, giving rise to novel polarization centers that resonate or vibrate under high-frequency EM fields, thereby enhancing localized polarization loss. Moreover, the spin polarization introduced by Mn–N_4_–C species and cross polarization induced by the helical structure substantially augment the EM loss capability. Ultimately, exemplary EMWA performance was achieved by crafting a gradient material. This innovative engineering approach to multiscale helical converters not only offers fresh insights into the quantitative mechanism of EM synergy but also opens up new horizons for the practical utilization of EMWA materials.

## Experimental Section

All experimental procedures, including synthesis methods, characterization techniques, and calculations, are detailed in the Supplementary Information.

### Synthesis of Antisense Strand

A solution containing 13.7 mmol of L-phenylglycine and 13.8 mmol of NaOH in 90 mL of deionized water and 30 mL of acetone was cooled in an ice-water bath. A total of 13.8 mmol of palmitoyl chloride and 13.8 mmol of NaOH were slowly added to the solution, which was then stirred for 12 h. The pH was adjusted to 1, and the antisense strand (AS) was obtained by filtration and purification in petroleum ether.

### Synthesis of Helical Converters

A solution containing 0.09 mmol of AS, 1.83 mmol of 3-aminophenol (3-AP), 0.09 mmol of (CH_3_COO)_2_Mn·4H_2_O and 8 mL of anhydrous methanol was prepared and heated to 60 °C. Subsequently, 62 mL of deionized water was added, followed by continuous stirring for 20 min and the addition of 200 µL of formalin aqueous solution (37 wt%) to induce polycondensation for 2 h. After vacuum filtration and drying for 12 h, the obtained precursors were annealed under a nitrogen atmosphere for 2 h at 800 °C at a rate of 2 °C min^−1^, denoted as HMC-8. Additionally, annealing was conducted at temperatures of 700 and 900 °C, resulting in materials denoted as HMC-7 and HMC-9, respectively. Following a similar synthesis process as for HMC-8, the amount of 3-AP was adjusted to 0.45 mmol to obtain SMC-8 and to 0.91 mmol to obtain SHMC-8, while the amount of (CH_3_COO)_2_Mn·4H_2_O was altered to 0.45 mmol to obtain LHMC-8 and to 0.9 mmol to obtain LMC-8.

### Characterization

The sample morphology was surveyed using field-emission scanning electron microscopy (FESEM; ZEISS Sigma 300) and transmission electron microscopy (TEM; Talos F2000X). Phase structures were determined using Powder X-ray diffraction (PXRD; Bruker D8 Advance A25) under CuKα radiation, while surface compositions were analyzed using X-ray photoelectron spectroscopy (XPS; Thermo Fischer ESCALAB Xi +). The degree of graphitization was assessed via Raman spectroscopy (Lab RAM HR800) in the range of 800 to 2000 cm^−1^ under 532 nm laser excitation. Vacancy defect information was obtained using the electron paramagnetic resonance (EPR; Bruker EMXplus-6/1). The EM characteristics of specimens in the 2–18 GHz range were analyzed using a vector network analyzer (Agilent PNA N5224A) employing the coaxial line method.

## Results and Discussion

### Synthesis of Helical Converters with Varied Geometrical Configurations

**Fig. 1:**
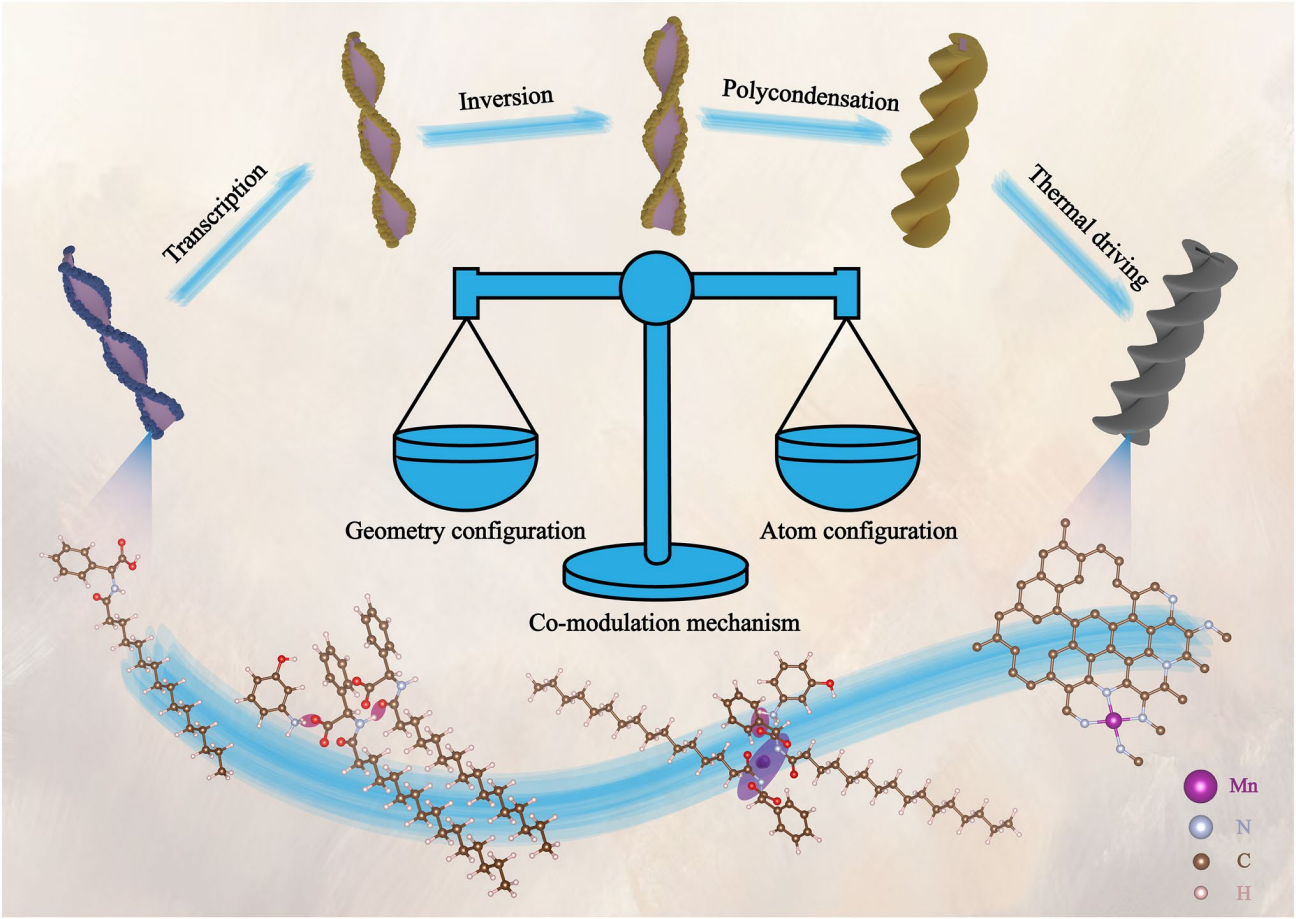
Synthesis process of the helical converters

Taking inspiration from the DNA transcription process, we synthesized helical converters using an in situ Mn/N co-doping approach, depicted in Fig. 1. (i) Synthesis of the AS: The AS was obtained via the acylation reaction of L-phenylglycine and palmitoyl chloride, resulting in a fibrous structure after self-assembly in a water–methanol solution [28]. (ii) Transcription process: The AS served as a template for constructing left-twisted nanohelices of 3-AP through both electrostatic interactions and the intermolecular hydrogen bonding between amide and carboxyl groups. (iii) Inversion process: The introduction of Mn^2+^ disrupted the original stacking pattern, leading to a completely new coordination pattern among Mn^2+^, carboxyl, and amide groups, thereby achieving helical inversion. (iv) Polycondensation and thermosetting process: The addition of formaldehyde induced polycondensation and a thermosetting reaction with 3-AP, forming a helical converter precursor. (v) External thermal driving process: The helical converters were obtained by annealing the precursor in a nitrogen atmosphere.

**Fig. 2:**
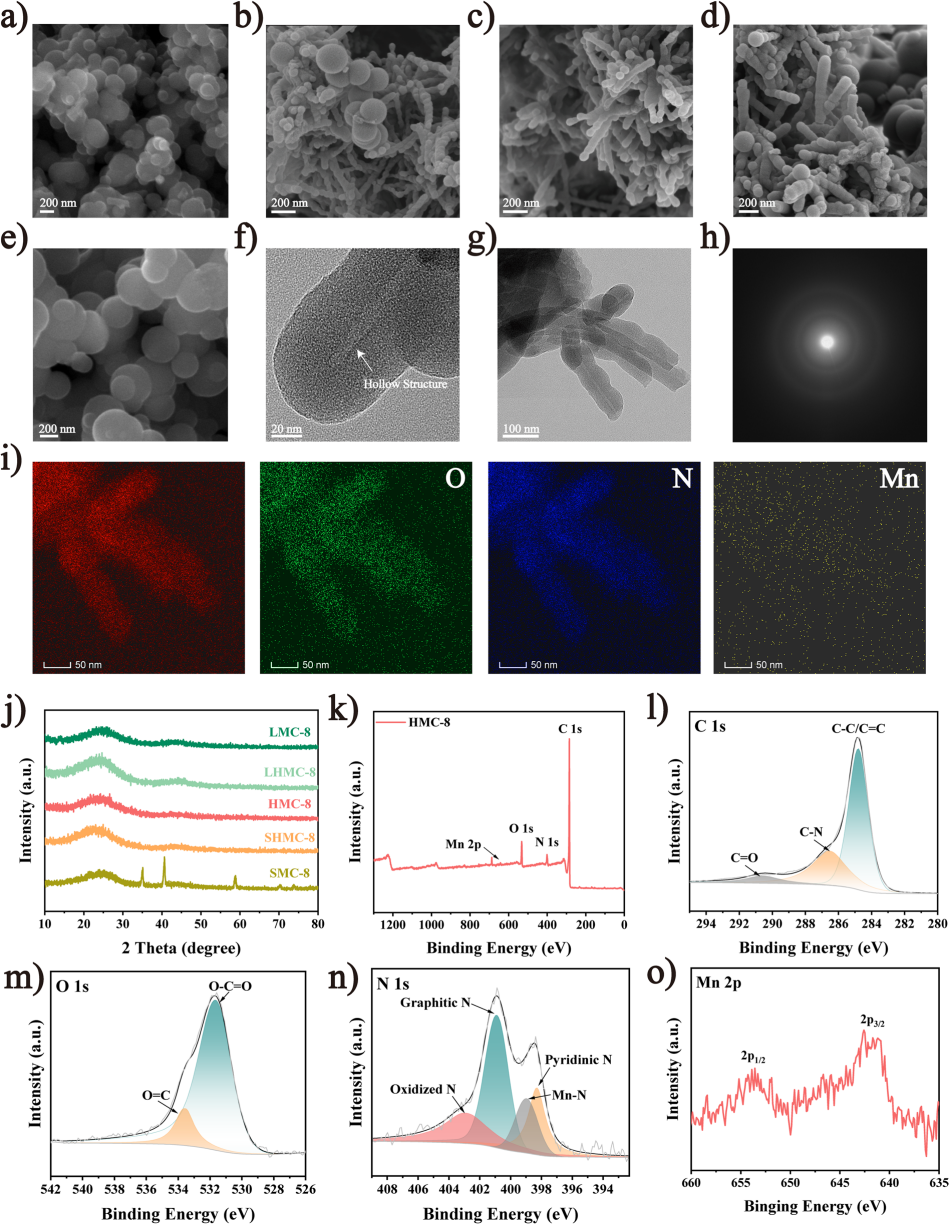
Structural characterization of helical converters. **a**–**e** FESEM images of SMC-8, SHMC-8, HMC-8, LHMC-8, and LMC-8 with a 200 nm scale. **f**–**h** TEM images and diffraction pattern of HMC-8. **i** EDS images of HMC-8 with the elements C, O, N, and Mn. **j** PXRD patterns of SMC-8, SHMC-8, HMC-8, LHMC-8, and LMC-8. **k**–**o** Full XPS survey spectra and high-resolution XPS spectra of C 1*s*, O 1*s*, N 1*s*, and Mn 2*p* of HMC-8

We observed that the ratio of Mn to 3-AP significantly influenced the geometry of the helical converters. Initially, keeping the Mn:3-AP ratio at 1:5 revealed the presence of small spheres with a diameter of approximately 171 nm, labeled SMC-8 (Figs. 2a and S2a). Subsequently, maintaining the Mn:3-AP ratio at 1:10 resulted in the coexistence of small spheres and anisotropic helical structures, denoted as SHMC-8 (Figs. 2b and S2b). Subsequently, keeping the amount of Mn constant while increasing that of 3-AP led to a Mn:3-AP ratio of 1:20, resulting in the appearance of only helical structures, named HMC-8 (Figs. 2c and S2c). Afterward, altering the Mn:3-AP ratio to 5:20 revealed the presence of large spheres with a diameter of approximately 299 nm alongside the helical structures, designated as LHMC-8 (Figs. 2d and S2d). Finally, varying the Mn:3-AP ratio to 10:20 caused only large spheres to be present, designated as LMC-8 (Figs. 2e and S2e), with the size of the spheres being comparable to those in 3-aminophenol formaldehyde resin derivatives (3-APFD, Fig. S2f). The formation of small spheres stemmed from AS not being encapsulated by 3-AP, thus tending to adopt a small spherical shape with reduced surface Gibbs free energy during external heat treatment. Conversely, excessive metal ions disrupted the electrostatic interaction between 3-AP and AS, leading to the formation of large-sized spheres [29]. Our results indicated that the optimal Mn:3-AP ratio was 1:20, forming a complete helical structure. TEM (Fig. 2f-g) revealed a distinct wavy shape characteristic of a hollow helical structure. Additionally, high-resolution TEM (HRTEM) images (Fig. S3) and diffraction patterns (Fig. 2h) exhibited an amorphous carbon nature, with lattice stripes not being regularly oriented and clear amorphous rings. Energy-dispersive X-ray spectroscopy (EDS) analysis (Fig. 2i) revealed a uniform distribution of elements, including C, O, N, and Mn, confirming successful Mn/N doping into carbon.

PXRD patterns of SMC-8, SHMC-8, HMC-8, LHMC-8, and LMC-8 are presented in Fig. 2j. SMC-8 exhibits five characteristic peaks at 34.9°, 40.5°, 58.7°, 70.2°, and 73.8°, which is attributed to (111), (200), (220), (311), and (222) planes of MnO [30]. This is due to the deficiency of N content. With the 3-AP content increasing, the two broad characteristic peaks observed at approximately 23.5° and 43.8° correspond to the (002) and (100) crystal planes of graphitized carbon. The intense diffraction peak at 23.5° indicates highly disordered carbon atoms, consistent with the HRTEM image. The peak at 43.8° may signify the presence of tiny crystalline domains formed during the high-temperature calcination process. The broadening of peaks suggests heteroatom doping and structural defects in the carbon materials [31–33]. Notably, no distinct diffraction corresponding to Mn is observed, indicating that Mn may be dispersed as single atoms on the material surface [34].

XPS was employed to examine the surface element composition and chemical environment of HMC-8 [35, 36]. The full XPS survey spectra (Fig. 2k) confirm the presence of C, N, O, and Mn. The high-resolution C 1*s* spectrum (Fig. 2l) displays peaks at 284.8, 286.5, and 289.5 eV, corresponding to C–C/C=C, C–N, C=O species, respectively, indicating N doping in carbon, which enhances conduction loss [37, 38]. The O 1*s* spectrum (Fig. 2m) exhibits peaks at 531.6 and 533.5 eV, attributed to O–C=O and O=C, respectively [39, 40]. The high-resolution N 1 s spectrum (Fig. 2n) shows peaks at 398.3, 398.9, 400.9, and 402.8 eV, corresponding to pyridinic N, Mn-N, graphitic N, and oxidized N species, respectively [41, 42]. Pyridinic N donates *p*-electrons to the π-conjugated system, while the N atom acts as a dipole, inducing dipole polarization [43]. The Mn 2*p* spectrum (Fig. 2o) exhibits peaks at 653.8 and 641.6 eV, indicating Mn 2*p*_1/2_ and Mn 2*p*_3/2_, respectively [44]. The results are consistent with the TEM findings, which indicate a lack of agglomeration, suggesting that the Mn is likely doped as a single atom in the materials.

**Fig. 3:**
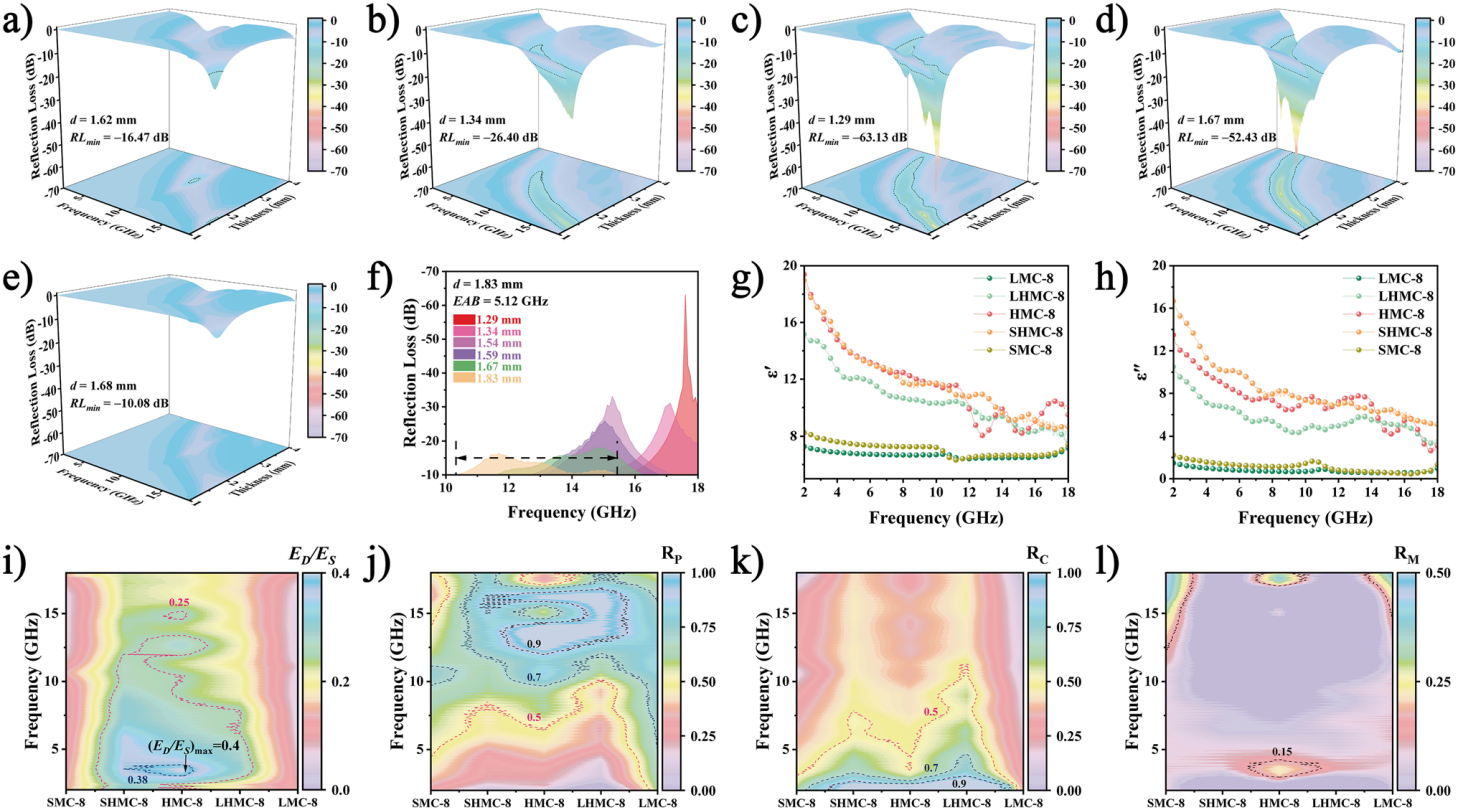
EMWA performance and EM quantitative analysis of helical converters. **a**–**e** 3D *RL* curves of SMC-8, SHMC-8, HMC-8, LHMC-8, and LMC-8, respectively. **f** EAB curves of HMC-8. **g**–**h** EM parameters and **i**–**l** *E*_*D*_*/E*_*S*_, *R*_*P*_, *R*_*C*_, and *R*_*M*_ curves of SMC-8, SHMC-8, HMC-8, LHMC-8, and LMC-8

The varying Mn:3-AP ratios significantly affect the EMWA property, as evidenced by minimum reflection loss (*RL*_*min*_) and EAB. Specifically, *RL*_*min*_ values of −16.47, −26.40, −63.13, −52.43, and −10.08 dB are achieved at thicknesses of 1.62, 1.34, 1.29, 1.67, and 1.68 mm for SMC-8, SHMC-8, HMC-8, LHMC-8, and LMC-8, respectively (Fig. 3a–e). EAB values of 0.72, 4.8, 5.12, 4.48, and 0.08 GHz are achieved at thicknesses of 2.85, 1.54, 1.83, 1.59, and 1.65 mm for SMC-8, SHMC-8, HMC-8, LHMC-8, and LMC-8, respectively (Figs. 3f and S6a–d). HMC-8, with its full helical structure, emerges as the most promising candidate, offering a light weight, thin profile, robust absorption capability, and broad absorption bandwidth.

EM parameters and loss capabilities were examined to understand the dissipation mechanism. In the 2–18 GHz frequency range, the *ε′* values for the SMC-8, SHMC-8, HMC-8, LHMC-8, and LMC-8 samples range from 8.2 to 6.3, 18.9 to 8.5, 19.3 to 8.0, 15.1 to 7.6, and 7.2 to 6.3, while the *ε′′* values vary from 2.2 to 0.4, 16.6 to 5.0, 13.4 to 2.6, 10.4 to 3.2, and 1.4 to 0.5, respectively (Fig. 3g–h). In general, *ε′* correlates with the multipolarization effect [45]. SHMC-8 and HMC-8 possess the largest *ε′* values, suggesting strong internal polarization ability, while SMC-8 exhibits the largest *ε′′* values owing to its shape anisotropy and nano-size effect [46]. The 3D Cole–Cole curves of all five samples in Fig. S8a–e indicate the Debye relaxation process. All the samples display multiple semicircles and long tails, attributed to strong polarization loss induced by Mn/N doping, defects, heterogeneous interfaces, and conduction loss from electron leaps and migrations in the conduction network [47].

Generally, dielectric loss and magnetic loss are discussed separately, which can obscure their interconnectedness. Helical materials possess a unique structure capable of generating cross polarization, which enhances EM loss. The impact of magnetic loss on EMWA performance cannot be overlooked. Consequently, we propose a comprehensive mechanism that considers the influence of magnetic loss, thereby further elucidating the dissipation mechanism. EMWA materials are known to convert EM energy into heat or other forms of energy [48–51]. When EMWs penetrate the material, the total energy (*E*_*0*_) divides into two components: the storage part (*E*_*S*_) involving the permittivity and permeability and the dissipation part (*E*_*D*_) involving the permittivity and permeability. Figure 3i shows the ratio of *E*_*D*_/*E*_*S*_, which represents the energy dissipation rate. The dissipation capacity of helical structure is greater than that of other structures. Specifically, HMC-8 with a full helical structure exhibits the strongest dissipation capacity. For the quantitative analysis of each loss, *R*_*P*_, *R*_*C*_, and *R*_*M*_ represent the transformation rate of polarization, conduction, and magnetic loss, respectively. As depicted in Fig. 3j–l, the polarization process primarily occurs in the medium- and high-frequency regions for all samples. Charge accumulation, increased interfaces, and cross polarization in the helical threaded structures substantially enhance *R*_*P*_, resulting in the observation of the highest *R*_*P*_ in HMC-8 [52]. Carriers migrate directionally and collide within the conduction network, converting EM energy into thermal energy. Conduction loss is mostly concentrated in the low-frequency regions owing to competitive effects with polarization loss [53]. Magnetic loss mainly occurs in both low- and high-frequency regions, with low-frequency occurrences attributed to natural resonances and high-frequency ones attributed to exchange resonances. Cross polarization induced by the helical structure optimizes magnetic loss, and it can be further enhanced by a similar structure. Thus, HMC-8 exhibits the highest *R*_*M*_.

Excellent EMWA materials necessitate optimal impedance matching (Fig. S9a–e). Compared to other samples, HMC-8 demonstrates superior impedance matching within a thickness of 2.00 mm, promoting EMW penetration. The attenuation constant (*α*) is another critical parameter for evaluating EMW loss ability (Fig. S9f). HMC-8 and SMC-8 exhibit higher *α* values, indicating stronger attenuation capability. However, HMC-8 can achieve strong EMWA over a wider frequency range because of superior impedance matching.

### Helical Converters with Different Graphite Domain Sizes

The graphite domain size closely correlates with EM parameters. To further optimize impedance matching and establish a correlation between graphite domain size and EMWA performance, HMC-7, HMC-8, and HMC-9 were obtained at various calcination temperatures. Notably, the annealing temperature does not considerably alter the geometric configuration, and the right-handed nanohelices are maintained (Fig. S12a–b).

**Fig. 4:**
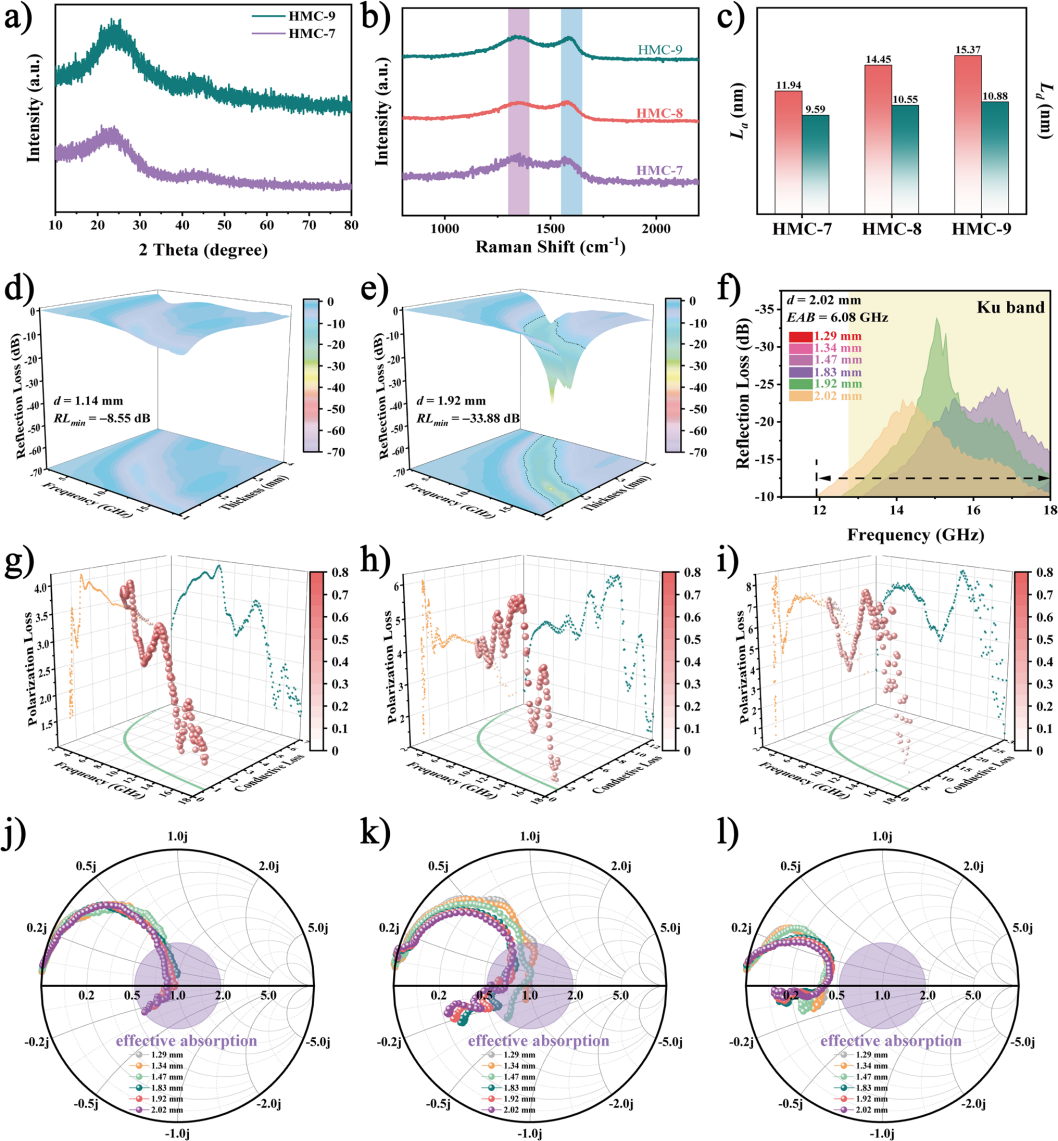
Structural characterization and EM property of helical converters with different graphite domain sizes. **a** PXRD patterns of HMC-7 and HMC-9. **b** Raman spectra and **c** *L*_*a*_ and *L*_*d*_ of HMC-7, HMC-8, and HMC-9. **d**–**e** 3D *RL* curves of HMC-9 and HMC-7. **f** EAB of HMC-7. **g**–**i** Polarization percentage and **j-l** Smith charts of HMC-7, HMC-8, and HMC-9

The PXRD patterns (Fig. 4a) of HMC-7 and HMC-9 are similar to those of HMC-8, indicating that variations in graphite domain sizes do not alter the phase composition of the material. To further investigate the extent of defects, Raman spectroscopy was employed (Fig. 4b). For carbon materials, the D peak at 1340 cm^−1^ and the G peak at 1580 cm^−1^ correspond to defect-induced structures and crystalline graphite structures, respectively [54]. The I_D_/I_G_ values for HMC-7, HMC-8, and HMC-9 are 1.61, 1.33, and 1.25, respectively. Note that all the I_D_/I_G_ values are greater than 1.00, indicating the amorphous nature of the carbon. Using these values and provided formulas (Eqs. S16-S18), we derived the graphite domain sizes (*L*_*α*_), defect distances (*L*_*d*_), and defect densities (*n*_*d*_) [55–57]. While the *L*_*α*_ values of the samples (Fig. 4c) gradually increase from 11.94 to 15.37 nm, the *L*_*d*_ values show a similar trend, increasing from 9.59 to 10.88 nm. Conversely, the *n*_*d*_ values slowly decrease (see Table S1 for details). These findings indicate that increasing the temperature enlarges the graphite domain size, thus increasing defect distances and enhancing overall conductivity. However, excessive conductivity can generate a skin effect, leading to impedance mismatch and reducing broadband absorption. Therefore, adequately regulating the graphite domain size is crucial for achieving broad absorption.

To assess the impact of different graphite domain sizes on EMWA performance, we evaluated the performance from 2 to 18 GHz (Fig. 4d–e). The *RL*_*min*_ value of HMC-9 only reaches −8.55 dB at a 1.14 mm thickness, failing to exhibit EMWA performance below −10 dB. In contrast, HMC-7 achieves −33.38 dB at a thickness of 1.92 mm. The EAB (Fig. 4f) of HMC-7 reaches 6.08 GHz at a 2.02 mm thickness, covering the entire Ku-band (12–18 GHz). Compared to HMC-8, HMC-7 has a broader EAB, and the absorption position shifts from the X/Ku band to the Ku band upon meticulous control of the graphite domain size.

The substantial differences in EMWA performance necessitate further explanation in terms of EM parameters. The *ε′* and *ε″* values of HMC-9 and HMC-7 are shown in Fig. S13a–b. Notably, all samples exhibit a similar trend: the *ε′* and *ε″* values decrease with increasing frequency, attributed to the dispersion effect induced by the polarized rotational hysteresis of high-frequency dipoles [58]. Additionally, the increase in permittivity is particularly evident with larger graphite domain sizes. In similar structures, the intensity of resonance peaks is closely related to the number of defects. Based on the Raman results, HMC-8 and HMC-9 have significantly fewer defects than HMC-7. Consequently, the resonance peaks pronounced in HMC-7 are attributed to the cumulative amplification effect produced by similar polarization [59].

To elucidate the dynamic behavior of dielectric loss, we fitted a modified Debye relaxation model using the least squares method [60, 61]. As shown in Fig. 4g-i, we quantify the contributions of *ε*_*p*_*"* and *ε*_*c*_*"* to dielectric loss and define *ε*_*p*_*"*/*ε"* as the polarization percentage. The larger and redder the balls, the greater the relative contribution of polarization loss. It is evident that the polarization loss in HMC-8 and HMC-7 is significantly greater than in HMC-9 owing to the numerous defects in HMC-8 and HMC-7. In contrast, HMC-9 exhibits the highest conduction loss owing to its graphite domain size being the largest.

Achieving suitable impedance matching entails ensuring that a larger portion of the input impedance (*Z*_*in*_) falls within the effective absorption area represented by the purple circle on the Smith charts (Fig. 4j–l). *Z*_*in*_ initially exhibits an inductive feature in the low-frequency region, transitioning into a capacitive feature as the frequency increases. Additionally, *Z*_*in*_ crosses the horizontal axis once, indicating resonance [62]. Notably, only the *Z*_*in*_ of HCM-7 has the most points within the purple circle at 2.02 mm, suggesting that HMC-7 boasts superior impedance matching characteristics at that thickness. Conversely, HMC-8 fails to fully access the purple region in the Ku band, with significantly fewer points within the circle compared to HMC-7. Furthermore, HMC-9 does not intersect the purple circle at all, indicating poor impedance matching behavior. These results align with those in Fig. S13c–d, further validating the superior impedance matching characteristics of HMC-7 with a moderate graphite domain size. Figure S13e reveals the calculated α values, with HMC-9 displaying the highest *α* values, with the worst impedance matching and weakest EMWA performance. Meanwhile, HMC-8 exhibits higher *α* values than HMC-7, suggesting greater attenuation ability. Furthermore, the maximum effective absorption integration area (*MEAIA*) is employed to characterize the practical application potential (Eq. S19). HMC-7 presents a significant advancement over HMC-8 (Fig. S13f), with its *MEAIA* encompassing 111% and 240% of *RL*_*min*_ and EAB_*max*_, respectively. These larger *MEAIA* values underscore the excellent EMWA characteristics of HMC-7, rendering it valuable for practical application.

### Mechanism Discussion

**Fig. 5:**
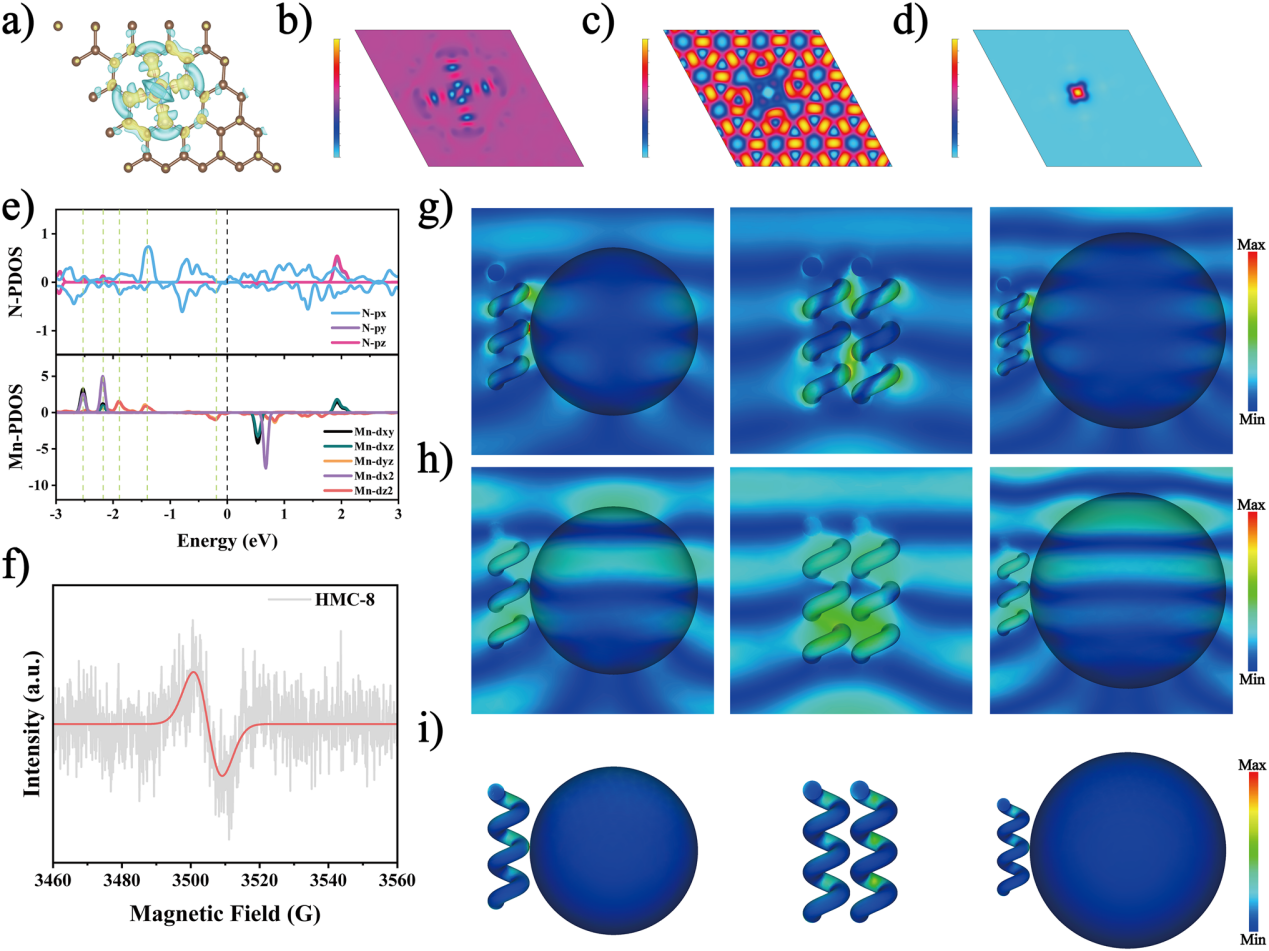
Mechanism of co-modulated atomic and geometric configurations. **a** 2D, **b** 3D charge density difference, **c** ELF, **d** spin density diagram, and **e** PDOS of Mn–N_4_–C species. **f** EPR of HMC-8. **g** Electric field distribution, **h** magnetic field distribution, and **i** power loss density of SHMC-8, HMC-8, and LHMC-8 at 17.6 GHz

Density functional theory (DFT) calculation elucidates the electron structure, EM loss mechanism, and structure–performance relationship of helical converters. In Fig. 5a–b, the charge density difference is depicted, where the yellow area indicates an increase in charge and the blue area represents a decrease, signifying the accumulation of rich charges around the Mn-N bonds. The distribution of positive and negative charges around Mn is nonuniform owing to differences in atom electronegativity, resulting in numerous electric dipoles. Bader charge analysis reveals that the Mn atom transfers 1.26 electrons to the N atom. Electron localization function (ELF) analysis (Fig. 5c) confirms the susceptibility of the electrons around Mn atoms to delocalization. Introducing Mn–N_4_–C species alters the localized electron structure, causing deviations in the intrinsic dipole moment and creating permanent electric dipoles. These dipoles resonate or vibrate under high-frequency EM fields, leading to relaxation processes and facilitating dielectric loss [63].

The electron structure information is further elucidated through density of states (DOS) calculation. As depicted in Fig. 5e, an overlap between the *d*-orbital of Mn and *p*-orbital of N occurs near the Fermi level, indicating *d–p* orbital coupling and the formation of hybridized orbitals, which further stabilizes the Mn–N_4_–C structure. The partial density of states (PDOS) image exhibits asymmetric behavior in different spin directions, while the spin density diagram (Fig. 5d) demonstrates that the abundant spin electrons of Mn form a high-spin state, enhancing spin polarization [64]. EPR curve (Fig. 5f) displays a robust resonance peak for HMC-8, indicating the formation of numerous dangling bonds and defects associated with unpaired electrons in the structure [65, 66]. The uneven distribution of charges induced by defects results in enhanced polarization loss.

To further analyze the interaction mechanism between geometric configuration and EMWs, finite element simulation was conducted at 2 GHz (Fig. S14a–c), 12 GHz (Fig. S15a-c), and 17.6 GHz (Fig. 5g–i), corresponding to low frequency, medium frequency, and the strongest absorption frequency, respectively. The electric field distribution is closely related to dielectric attenuation. HMC-8 exhibits the strongest interface polarization, primarily occurring in the helical threaded structure, while the interface polarization of SHMC-8 and LHMC-8 mainly occurs at the interfaces between the helical and spherical structures. The discontinuous interface distribution of the helical structure alters the charge accumulation and enhances interface polarization. Additionally, cross polarization induced by the helical structure enhances magnetic loss ability. The power loss density is closely related to the attenuation capability [67]. The helical structure has a large power attenuation area, further demonstrating its superior loss capability.

### Practical Application

**Fig. 6:**
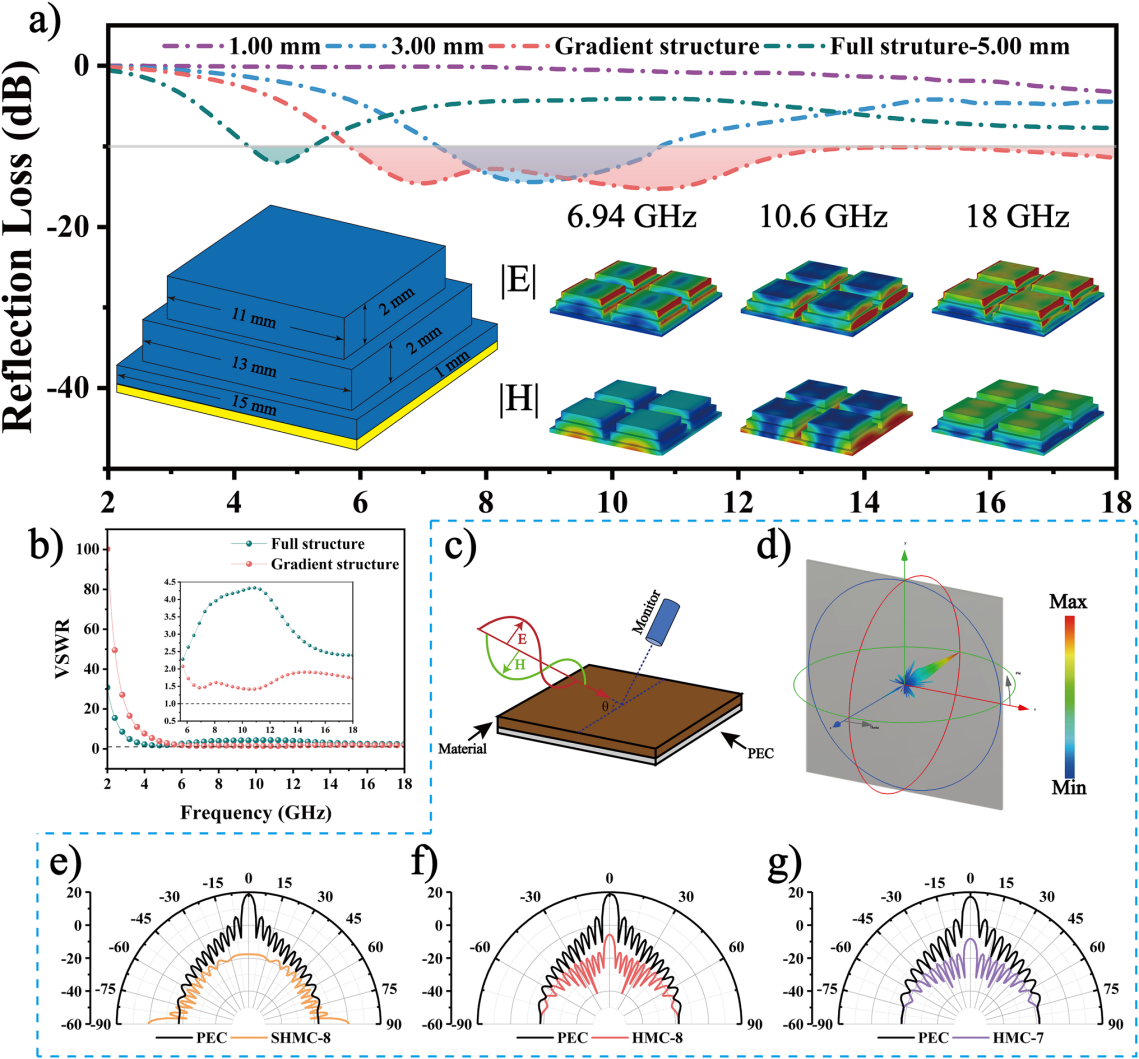
Practical application. **a** Gradient structure design of HMC-7 to enhance EMWA performance. **b** VSWR comparison between gradient and full structure. **c** Schematic of RCS simulation. **d** 3D RCS visualization of SHMC-8. **e**–**g** RCS presented in polar coordinate system for SHMC-8, HMC-8, and HMC-7

Single-layer coating materials, also known as fully structured materials, require the operating frequency to match the quarter wavelength (λ/4). The actual thickness of the six samples is matched to the simulated thickness (Eq. S20). With an increase in thickness, the absorption peaks of the *RL* gradually shift to lower frequencies (Fig. S17) [68]. To operate at different frequencies, the thickness of the single-layer coating material must be adjusted, which limits its practical application. The ingenious design of a gradient structure in metamaterials enables multiple EM responses, resulting in ultrabroadband EMW absorption. Adjusting the thickness of each layer in the gradient multilayer structure facilitates multiple interferences of λ/4 waves with each other, causing the generation of multiple EMWA peaks. These peaks are superimposed in the overall gradient structure, realizing ultrabroadband absorption. The gradient metamaterial extends the EAB of HMC-7 to 12.16 GHz (5.84–18 GHz), covering 67.5% of the entire band (2–18 GHz). As shown in Fig. 6a, the electric field at 6.94 GHz is predominantly distributed at the top, while the magnetic field is concentrated at the bottom, with a phase difference close to π/2. Standing waves are formed inside the material, indicating that interference dominates at this frequency. The attenuation at 10.6 GHz is localized mainly at the edges of the material, indicating that resonance dominates in the periodic structure. A very small absorption peak at 18 GHz is caused by the internal λ/4 interference of the material [69]. Furthermore, the electric field loss significantly exceeds the magnetic field loss because of the strong dielectric loss characteristic of the material, and the loss regions are mainly concentrated between the layers. Power loss (Fig. S18a–c) occurs in the bottom and top regions of the material. The voltage standing wave ratio (VSWR) is a crucial metric for evaluating impedance matching [70]. As displayed in Fig. 6b, in the 2–18 GHz range, VSWR values of the gradient structure are closer to 1 than those of the full structure, indicating better impedance matching of the gradient structure. Thus, this gradient structure is conductive to the realization of broadband absorption and further meets practical application requirements.

The radar cross section (RCS) is employed to simulate the far-field EM response, mirroring real-world conditions (Eq. S21) [71, 72]. In Fig. 6c, the samples are depicted as coated on a square (200 × 200 mm^2^) perfect electrical conductor (PEC) plate [73]. These plates, each bearing one of the seven samples, are positioned in the X-O-Y plane, with the angle of the EMWs relative to the X-O-Y plane denoted as θ. An angle of 0° indicates vertical incidence of EMWs onto the plates. The maximum RCS reduction values are 16.90 and 12.28 dB m^2^ for HMC-9 and LHMC-8, respectively (Fig. S19g–h). Meanwhile, SHMC-8, HMC-8, HMC-7, SMC-8, and LMC-8 (Figs. 6e–g and S19i–j) all demonstrate outstanding radar stealth performance, with maximum RCS reduction values of 36.4, 35.19, 33.71, 32.04, and 31.03 dB m^2^, respectively. The excellent RCS absorption performance indicates that the helical converters are well-suited for EMW energy harvesting [74]. This provides further evidence of the promising application potential of helical converters.

**Fig. 7:**
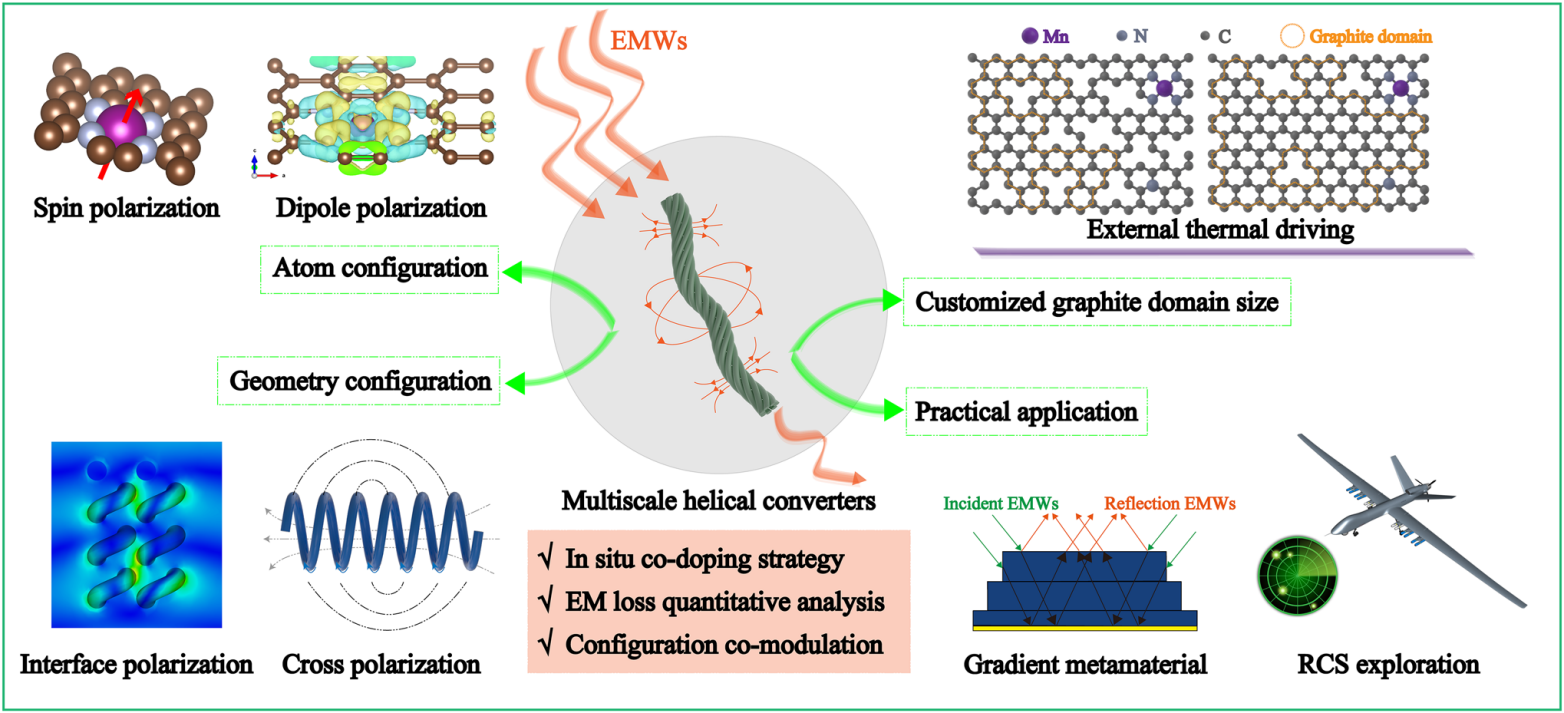
EMWA mechanism of the helical converters

Based on the DFT calculation and EM simulation results described above, the EMWA mechanism of the helical converters is systematically illustrated in Fig. 7. The exceptional EMWA performance primarily arises from the synergistic interaction between the co-modulation of atomic and geometric configurations and the macroscopic size design. At the atomic scale, the formation of Mn–N_4_–C species and defects induces alterations in the electronic structure near the Fermi level. This facilitates local charge rearrangement, leading to the creation of new polarization centers and reinforcing polarization loss. Moreover, the presence of abundant valence electrons of Mn fosters the formation of high-spin states, thereby enhancing spin polarization. At the microscopic scale, the intertwined network established by the distinctive helical configuration facilitates the ingress of EMWs, optimizing impedance matching and broadening the transmission path of EMWs, thus enhancing attenuation capability. This unique configuration not only alters the EM distribution at interfaces to bolster interface polarization but also engenders cross polarization, thereby augmenting EM loss capability. The formation of graphite domains through high-temperature calcination promotes the construction of conduction networks, thereby enhancing conduction loss, which converts EMW energy into dissipated thermal energy [75]. At the macro scale, a gradient structure is devised by adjusting the thickness of each layer to maximize impedance optimization and achieve compatibility with free space. Concurrently, the generation of multiple λ/4 attenuation modes within the gradient structure facilitates edge diffraction and resonance among adjacent unit cells, effectively dissipating EMWs. This multiscale design approach furnishes a novel dissipation mechanism for EMWA, enabling the realization of ultrabroadband EMWA for practical application.

## Conclusions

Multiscale helical converters were synthesized via the in situ metal–nonmetal co-doping of helical carbon nanotubes. The proposed co-modulation of atomic and geometric configurations established a quantitative link between configuration and EMW loss mechanism. Furthermore, the Mn–N_4_–C configuration induced orbital coupling and spin polarization, while the helical configuration generated cross polarization, enhancing EMW attenuation and absorption. Consequently, HMC-8, featuring Mn–N_4_–C and full helical configurations, exhibited exceptional EMWA performance with an *RL*_*min*_ value of −63.13 dB at an ultralow thickness of 1.29 mm. Additionally, external thermal driving processes modulated EMWA performance, yielding a broad EAB of 6.08 GHz. DFT calculation and EM simulation elucidated the EMWA mechanism, guiding the design of an ultrabroad EMWA metamaterial with an EAB of 12.16 GHz. Furthermore, far-field EM responses exhibited significant reductions in RCS, with a maximum reduction value of 36.4 dB m^2^. This systematic study introduces a novel concept for preparing high-performance EMWA materials and paves the way for practical application.

## Supplementary Information

Below is the link to the electronic supplementary material.

Supplementary file1 (DOCX 415004 KB)
