# Supplementary material for: Designing Electronic Structures of Multiscale Helical Converters for Tailored Ultrabroad Electromagnetic Absorption
Source: Nanomicro Lett. 2024 Sep 26;17:20. doi: 10.1007/s40820-024-01513-2 (PMC11448510; doi:10.1007/s40820-024-01513-2)
Supplement: Supplementary file 1 — Supplementary file1 (DOCX 415004 KB) [file 40820_2024_1513_MOESM1_ESM.docx]

Supporting Information for

**Designing Electronic Structures of Multiscale Helical Converters for Tailored Ultrabroad Electromagnetic Absorption**

Zhaobo Feng^1^, Chongbo Liu^1,^ *, Xin Li^1^, Guangsheng Luo^2^, Naixin Zhai^2^, Ruizhe Hu^1^, Jing Lin^1^, Jinbin Peng^1^, Yuhui Peng^3,^ *, Renchao Che^4,^ *

^1^ Key Laboratory of Jiangxi Province for Persistent Pollutants Control and Resources Recycle, School of Environmental and Chemical Engineering, Nanchang Hangkong University, Nanchang 330063, P. R. China

^2^ School of Physics and Materials, Nanchang University, Nanchang, 330031, P. R. China

^3^ Key Laboratory of Nondestructive Testing, Ministry of Education, Nanchang Hangkong University, Nanchang 330063, P. R. China

^4^ Laboratory of Advanced Materials, Shanghai Key Lab of Molecular Catalysis and Innovative Materials, Academy for Engineering & Technology, Fudan University, Shanghai 200438, P. R. China

*Corresponding authors. E-mail: [cbliu2002@163.com](mailto:cbliu2002@163.com) (Chongbo Liu); [pengyuhui@nchu.edu.cn](mailto:pengyuhui@nchu.edu.cn) (Yuhui Peng); [rcche@fudan.edu.cn](mailto:rcche@fudan.edu.cn) (Renchao Che)

**S1 Experimental Section**

L-phenylglycine, (CH_3_COO)_2_Mn·4H_2_O, and anhydrous methanol were purchased from Bide Pharmatech Co., Ltd. (Shanghai, China). Palmitoyl chloride, NaOH, acetone, formalin aqueous solution (37 wt%), petroleum ether, and 3-aminophenol (3-AP) were purchased form Aladdin Biochemical Technology Co., Ltd. (Shanghai, China). All chemicals were used without further purification.

**S1.1 Synthesis of HM, SHM, and LHM**

A solution containing 0.09 mmol of the antisense strand (AS), 1.83 mmol of 3-AP, 0.09 mmol of (CH_3_COO)_2_Mn·4H_2_O were added into 8 mL of anhydrous methanol, and heated to 60 °C. Subsequently, 62 mL of deionized water was added, followed by continuous stirring for 20 min, and the product was denoted as HM. Following a similar synthesis process as for HM, the amount of 3-AP was adjusted 0.91 mmol, and this product was named as SHM; while the amount of (CH_3_COO)_2_Mn·4H_2_O was changed to 0.45 mmol, which was denoted as LHM.

**S1.2 Synthesis of 3-APFD and HC-8**

3-AP (1.83 mmol) was completely dissolved in anhydrous methanol (8 mL) at 60 °C. Subsequently, deionized water (62 mL) was added to the above solution. After continuous stirring for 20 minutes, 200 µL formalin aqueous solution (37 wt%) was added to induce polycondensation reaction, continuing for 2 h. The precursors were gathered through vacuum filtration and dried for 12 h, subsequently, were annealed under nitrogen atmosphere for 2 h at 800 °C at a rate of 2 °C/min, denoted as 3-APFD. Following a similar synthesis process as for 3-APFD, the AS (0.09 mmol) was added, which was named as HC-8.

**S1.3 Characterization**

^1^H-Nuclear magnetic resonance (^1^H-NMR) spectra were recorded on a Bruker Avance III HD 500 MHz NMR spectrometer. The functional groups were detected by Fourier transform infrared spectroscopy (FT-IR; Bio-Rad FTS-40). The gradient mematerial, radar cross section (RCS) were simulated using computer simulation technology (CST Studio Suite 2022).

**S1.4 Electromagnetic (EM) Measurements**

The samples and paraffin were mixed in a certain proportion (the samples account for 30 wt%), and the coaxial ring with an inner diameter of 3.04 mm and an outer diameter of 7 mm was processed by the mold. As an electromagnetic wave absorption (EMWA) material, reflection loss (*RL*) and attenuation constant (*α*) are the important evaluation indexes. According to transmission line theory, *RL* and *α* can be calculated by the following formula [S1-S3]:

$Z_{in}=\sqrt{\frac{\mu_{r}}{\varepsilon_{r}}}tanh\left[ j(2\pi fd/c)\sqrt{\mu_{r}\varepsilon_{r}} \right]$	(Eq. S1)

$RL=20log\left| {(Z}_{in}-1)/{(Z}_{in}+1) \right|$	(Eq. S2)

$\alpha=\frac{\sqrt{2}\pi f}{c}\sqrt{(\mu^{''}\varepsilon^{''}-\mu^{'}\varepsilon^{'})+\sqrt{{{(\mu}^{'}\varepsilon^{''}+\mu^{''}\varepsilon^{'})}^{2}+{{(\mu}^{''}\varepsilon^{''}-\mu^{'}\varepsilon^{'})}^{2}}}$	(Eq. S3)

Where *Z_in_* is the normalized input impedance of EMWA materials, *Z_0_* is the free space impedance; $\varepsilon_{r}$ and $\mu_{r}$ represent complex permittivity and complex permeability, respectively; *d* is the thickness of the EMWA materials; *f* is the frequency of electromagnetic waves (EMWs). The Debye relaxation formula is an important means of studying polarization effect as follows [S4-S6]:

${(\varepsilon'-\frac{\varepsilon_{s}+\varepsilon_{\infty}}{2})}^{2}+{(\varepsilon")}^{2}={(\frac{\varepsilon_{s}-\varepsilon_{\infty}}{2})}^{2}$	(Eq. S4)

$\varepsilon'=\varepsilon_{\infty}+\frac{\varepsilon_{s}-\varepsilon_{\infty}}{1+\omega^{2}\tau^{2}}$	(Eq. S5)

$\varepsilon''=\frac{\varepsilon_{s}-\varepsilon_{\infty}}{1+\omega^{2}\tau^{2}}\omega\tau+\frac{\sigma}{\omega\varepsilon_{0}}=\varepsilon_{p}^{''}+\varepsilon_{c}^{''}$	(Eq. S6)

Where $\varepsilon_{\infty}$ represents the relative dielectric permittivity at infinite frequency and $\varepsilon_{s}$ is the static dielectric permittivity, *ω* is the angular frequency, *τ* is the relaxation time and *σ* is the electric conductivity.

According to basic laws of dielectric and magnetic physics, the absorbed incident EM energy (*E_0_*) can be divided into two parts and expressed as follows [S7-S9]:

$E_{0}=E_{S}+E_{D}$	(Eq. S7)

where $E_{S}$ and $E_{D}$ represent the energy of storage part and conversion part, respectively.

$E_{S}$ is contributed by the real part of the permittivity and permeability, which can be explained as follows:

$E_{S}=\pi f\varepsilon^{'}E_{0}^{2}+\pi f\mu^{'}H_{0}^{2}$	(Eq. S8)

$E_{D}$ is contributed by the imaginary part of the permittivity and permeability, which can be expressed as follows:

$E_{D}=\pi f\varepsilon^{''}E_{0}^{2}+\pi f\mu^{''}H_{0}^{2}$	(Eq. S9)

where *E_0_* and *H_0_* are the electric and magnetic field intensity amplitude of EMWs, respectively.

As the loss can be divided into polarization ($\varepsilon_{p}^{''}$), conduction ($\varepsilon_{c}^{''}$), and magnetic loss ($\mu^{''}$):

$E_{P}=\pi f\varepsilon_{p}^{''}E_{0}^{2}$	(Eq. S10)

$E_{C}=\pi f\varepsilon_{c}^{''}E_{0}^{2}$	(Eq. S11)

$E_{M}=\pi f\mu^{''}H_{0}^{2}$	(Eq. S12)

where $E_{P}$, $E_{C}$ and $E_{M}$ represent conversion energy contributed by polarization loss, conduction loss and magnetic loss, respectively.

The transformation rate of converted EMWs derived from polarization (*R_P_*) and conduction (*R_C_*), and magnetic loss (*R_M_*) can be expressed as follows:

$R_{P}=\frac{E_{P}}{E_{D}}$	(Eq. S13)

$R_{C}=\frac{E_{C}}{E_{D}}$	(Eq. S14)

$R_{M}=\frac{E_{M}}{E_{D}}$	(Eq. S15)

**S1.5 Graphite domain size calculations**

Graphite domain size (*L_α_*), defects distance (*L_d_*), and defect density (*n_d_*) can be expressed as follows [12, 13]:

$L_{\alpha}\left( nm \right)=\left( 2.4\times{10}^{-10} \right)\times\lambda^{4}\times{(I_{D}/I_{G})}^{-1}$	(Eq. S16)

$L_{d}\left( nm \right)=\sqrt{\left( 1.85\times{10}^{-9} \right)\times\lambda^{4}\times{(I_{D}/I_{G})}^{-1}}$	(Eq. S17)

$n_{d}\left( nm \right)=\left( 1.85\times{10}^{22} \right)\times\lambda^{-4}\times(I_{D}/I_{G})$	(Eq. S18)

**S1.6 Maximum effective absorption integration area (*MEAIA*)**

The *MEAIA* is used to characterize its practical application potential:

$(\int RL(\leq-10 dB)df){(t)}^{-1}$	(Eq. S19)

where *t* represents the thickness of the thickness.

**S1.7 EM simulation in CST**

A template of periodic structures with metamaterial-full structure workflow was chosen for simulation. Frequency domain solver was used in this simulation process, the solving frequency is set to 2-18 GHz. The excitation type set to plane wave excitation, and the incident direction of EMW was vertical to the model. The tetrahedral mesh type was used to mesh these models.

**S1.8 Quarter-wavelength theory**

The relation between the *RL*, thickness, and frequency can be obtained using the quarter-wavelength model. The simulated thickness (*t_m_*) of an absorber at 2–18 GHz is estimated as follows:

$t_{m}=nc/(4f_{m}\sqrt{|\mu_{r}||\varepsilon_{r}|})$	(Eq. S20)

where t_m_, n, and f_m_ denote the simulated thickness, the refractive index of the material, and the matched frequency, respectively.

**S1.9 RCS Simulation**

The actual far-field response of the material was simulated using CST Studio Suite 2022. The material was modeled as a two-layer square model with a metallic base plate and an absorber layer, where the size of the square was 200 mm × 200 mm, the thickness of the absorber layer was x mm (x corresponds to the thickness of *RL_min_* for each set of samples), and the thickness of the bottom perfectly conducting layer (PEC) was set to 2.0 mm. The model was placed in the X-O-Y plane, and the EMWs were incident from the positive direction of Z-axis. Adjusting the incident angle θ (-90°-90°) of EMWs while keeping the polarization angle φ constant, the RCS value can be expressed by *S* (area of the model), *λ* (wavelength of the EMW), *𝐸_𝑠_* (electric field strength) and *𝐸_𝑖_* (incident wave) [S10, S11].

$RCS\left( {dBm}^{2} \right)=10\log\left( \left( \frac{4\pi S}{\lambda^{2}} \right)\left| \frac{E_{s}}{E_{i}} \right| \right)^{2}$	(Eq. S21)

**S2 Results and Discussion**

**S2.1 FESEM of the AS, AS/3-AP, and HM**

**Fig. S1** FESEM of **a** AS, **b** AS/3-AP, and **c** HM

**S2.2 FESEM of the varied geometric configurations**

**Fig. S2** FESEM of **a** SMC-8, **b** SHMC-8, **c** HMC-8, **d** LHMC-8, **e** LMC-8 and **f** 3-APFD

**S2.3 HRTEM of the HMC-8**

**Fig. S3** HRTEM image of HMC-8

**S2.4. XPS of SHMC-8 and LHMC-8**

**Fig. S4 a** Full XPS survey spectra of SHMC-8 and LHMC-8, **b** N content and Mn content of SHMC-8, HMC-8, and LHMC-8

**S2.5 ^1^H-NMR and FT-IR of AS, AS/3-AP, HM, SHM and LHM**

To explore the chemical structure and coordination information of AS, AS/3-AP, HM, SHM and LHM, ^1^H-NMR and FT-IR were performed (Figs. S4a-b). The ^1^H-NMR spectra of AS was well-matched with the prediction of ChemDraw, implying the successful synthesis of AS. AS: ^1^H-NMR (500 MHz, DMSO-d6): δ=8.55 (s, 1H; CONHCH), 7.70-7.00 (m, 5H; Ph), 5.37 (d, J = 7.3 Hz, 1H; NHCHCOOH), 2.18 (t, J = 7.3 Hz, 2H; CH_2_CH_2_CONH), 1.48 (t, J = 7.6 Hz, 2H; CH_2_CH_2_CONH), 1.37-1.12 (m, 24H; alkyl), 0.85 (t, J = 6.7 Hz, 3H; CH_3_CH_2_).

The FT-IR showed that the new peaks did not appear in the AS/3-AP compound, suggesting that 3-AP was adsorbed on the surface of AS by electrostatic interaction. However, the addition of Mn ions significantly changed the coordination pattern, the C=O stretching vibration in carboxylate disappears at 1726 cm^-1^ and the new asymmetric stretching vibration of carboxylates appears at 1588 cm^-1^ and 1568 cm^-1^. Meanwhile, the amide I and amide II bands shifted to low wavenumbers, with a significant decrease in intensity. The transmittance of the peaks assigned as N-H stretching vibrations also decreased. This observation implied that Mn^2+^ coordinated with carboxyl and amide units. Further, an increase in the amount of Mn^2+^ did not change the characteristic peaks of the FT-IR spectrum, suggesting that the amount of Mn^2+^ was excessive relative to the AS amount at this time, which also proved that the optimal ratio of Mn^2+^ to AS was 1:1. AS, FT-IR (KBr): 3292 (s) cm^−1^ (N-H, amide A), 1648 (vs) cm^−1^ (CO, amide I), 1535 (vs) cm^−1^ (N-H, amide II).

**Fig. S5** **a** ^1^H-NMR of AS. **b** FT-IR of AS/3-AP, HM, SHM, LHM

**S2.6 EAB of varied geometric configurations**

**Fig. S6** EAB curves of **a** SMC-8, **b** SHMC-8, **c** LHMC-8, and **d** LMC-8

**S2.7 Electromagnetic parameters of varied geometric configurations**

**Fig. S7** **a-b** EM parameters of SMC-8, SHMC-8, HMC-8, LHMC-8, and LMC-8

**S2.8 3D Cole-Cole curves of varied geometric configurations**

**Fig. S8** 3D Cole–Cole curves of **a** SMC-8, **b** SHMC-8, **c** HMC-8, **d** LHMC-8, and **e** LMC-8

**S2.9 The impedance matching and the attenuation constant of varied geometric configurations**

**Fig. S9** **a-e** The impedance matching and **f** the attenuation constant of SMC-8, SHMC-8, HMC-8, LHMC-8, and LMC-8

**S2.10 The electromagnetic characteristic of 3-APFD and HC-8**

The *RL_min_* of 3-APFD is only −5.13 dB at a thickness of 1.78 mm because of the absence of the helical structure, which results in poor impedance matching and attenuation constant (**Fig. S10**).

**Fig. S10** **a** 3D *RL* curve, **b** EM parameters, **c** the impedance matching and **d** the attenuation constant of 3-APFD

HC-8 and HMC-8 share the same geometry configuration but differ in atom configuration. HC-8, lacking the Mn-N_4_-C configuration, only achieves an *RL_min_* of −36.58 dB at 1.51 mm and an EAB of 3.92 GHz at 1.7 mm. The permittivity and attenuation constant of HC-8 are significantly weaker than those of HMC-8 because of the absence of orbital coupling and spin polarization caused by the Mn-N_4_-C configuration. Thus, the EMW attenuation ability decreases dramatically, indicating that the internal atomic configuration is crucial in determining the EM properties of the material, even with the same geometry configuration.

**Fig. S11** **a** 3D *RL* curve, **b** EM parameters, **c** the impedance matching and **d** the attenuation constant of HC-8

**S2.11 FESEM of different graphite domain sizes**

**Fig. S12** FESEM images of **a** HMC-7 and **b** HMC-9

**S2.12 Graphite domain sizes**

**Table S1** The graphite domain sizes, defects distance, and defect density

| Sample | *L_α_* | *L_d_* | *n_d_* |
| --- | --- | --- | --- |
| HMC-7 | 11.94 nm | 9.59 nm | 3.71×10^11^ cm^-2^ |
| HMC-8 | 14.45 nm | 10.55 nm | 3.07×10^11^ cm^-2^ |
| HMC-9 | 15.37 nm | 10.88 nm | 2.88×10^11^ cm^-2^ |

**S2.13 Electromagnetic characteristic of different graphite domain sizes**

**Fig. S13** EM parameters and the impedance matching of **a-c** HMC-9, **b-d** HMC-7, respectively. **e** Attenuation constant of HMC-9 and HMC-7. **f** *MEAIA* of HMC-8 and HMC-7

**S2.14 Electromagnetic simulations**

**Fig. S14** **a** The electric field distribution, **b** the magnetic field distribution, and **c** the power loss density of HMC-8, SHMC-8 and LHMC-8 at 2 GHz

**Fig. S15** **a** The electric field distribution, **b** the magnetic field distribution, and **c** the power loss density of HMC-8, SHMC-8 and LHMC-8 at 12 GHz

Cross polarization was further verified by comparing the electric field loss strength and magnetic field loss strength of different structures. Two different models, the helical and spherical models, were simulated using the same electromagnetic parameters. As shown in **Figs. S16a-d**, the electric field and magnetic field loss strengths of the helical structure are approximately 4 and 1.7 times those of the spherical model, respectively. This is because the discontinuity of the boundary of the helical structure prevents the generated electromagnetic field components from offsetting each other. The stronger attenuation demonstrates that cross polarization is present, clearly showing that the geometric configuration plays a decisive role in electromagnetic loss under the same electromagnetic parameters.

**Fig. S16** Electric field and magnetic field simulation of **a-b** helical structure and **c-d** sphere structure at 17.6 GHz

**S2.15 Quarter-wavelength theory**

**Fig. S17** Frequency dependence of variation of *RL* values and simulation thickness of **a** SMC-8, **b** SHMC-8, **c** HMC-8, **d** LHMC-8, **e** LMC-8, and **f** HMC-7

**S2.16 Metamaterial simulations**

**Fig. S18** Power loss density of the gradient metamaterial at **a** 6.9 GHz, **b** 10.8 GHz, and **c** 18 GHz

**S2.17 RCS simulations**

**Fig. S19** **a-f** 3D RCS of HMC-7, HMC-8, HMC-9, SMC-8, LMC-8, and LHMC-8. **g-j** The 2D RCS of HMC-9, LHMC-8, SMC-8, and LMC-8

**Supplementary References**

[S1] N. Qu, G. X. Xu, Y. K. Liu, M. He et al., Multi-scale design of metal-organic framework metamaterials for broad-band microwave absorption. Adv. Funct. Mater. (2024). <https://doi.org/10.1002/adfm.202402923>
[S2] Z. Zheng, Y. Chen, H. Liu, H. Lin, H. Zhao et al., Facile fabrication of NiFe_2_O_4_-FeNi/C heterointerface composites with balanced magnetic-dielectric loss for boosting electromagnetic wave absorption. Chem. Eng. J. **481**, 148224 (2024). https://doi.org/10.1016/j.cej.2023.148224
[S3] F. Pan, X. Wu, D. Batalu, W. Lu, H. Guan, Assembling of low-dimensional aggregates with interlaminar electromagnetic synergy network for high-efficient microwave absorption. Adv. Powder Mater. **2**, 100100 (2022). https://doi.org/10.1016/j.apmate.2022.100100
[S4] J. Zhou, D. Lan, F. Zhang, Y. Cheng, Z. Jia et al., Self-assembled MoS_2_ cladding for corrosion resistant and frequency-modulated electromagnetic wave absorption materials from X-band to Ku-band. Small **19**, 2304932 (2023). https://doi.org/10.1002/smll.202304932
[S5] H. Xu, X. Yin, M. Li, F. Ye, M. Han et al., Mesoporous carbon hollow microspheres with red blood cell like morphology for efficient microwave absorption at elevated temperature. Carbon **132**, 343-351 (2018). <https://doi.org/10.1016/j.carbon.2018.02.040>
[S6] F. Wang, W. Gu, J. Chen, Q. Huang, M. Han et al., Improved electromagnetic dissipation of Fe doping LaCoO_3_ toward broadband microwave absorption. J. Mater. Sci. Technol. **105**, 92-100 (2022). https://doi.org/10.1016/j.jmst.2021.06.058
[S7] Q. Wang, B. Niu, Y. Han, Q. Zheng, L. Li et al., Nature-inspired 3D hierarchical structured "vine" for efficient microwave attenuation and electromagnetic energy conversion device. Chem. Eng. J. **452**, 139042 (2023). <https://doi.org/10.1016/j.cej.2022.139042>
[S8] J. Shu, X. Huang, M. Cao, Assembling 3D flower-like Co_3_O_4_-MWCNT architecture for optimizing low-frequency microwave absorption. Carbon **174**, 638-646 (2021). <https://doi.org/10.1016/j.carbon.2020.11.087>
[S9] J. Shu, M. Cao, M. Zhang, X. Wang, W. Cao et al., Molecular patching engineering to drive energy conversion as efficient and environment-friendly cell toward wireless power transmission. Adv. Funct. Mater. **30**, 1908299 (2020). <https://doi.org/10.1002/adfm.201908299>
[S10] T. Xu, J. Li, D. Zhao, Z. Zhang, X. Li et al., Synchronous manipulation of heterointerfaces and atomic hybrids in bimetallic max phase composites for advanced electromagnetic wave absorption. Compos. Part B Eng. **271**, 111148 (2024). https://doi.org/10.1016/j.compositesb.2023.111148
[S11] X. Li, R. Hu, Z. Xiong, D. Wang, Z. Zhang et al., Metal-organic gel leading to customized magnetic‑coupling engineering in carbon aerogels for excellent radar stealth and thermal insulation performances. Nano-Micro Lett. **16**, 42 (2024). <https://doi.org/10.1007/s40820-023-01255-7>
[S12] J. Tao, L.Xu, H. Jin , Y. Gu, J. Zhou et al., Selective coding dielectric genes based on proton tailoring to improve microwave absorption of MOFs. Adv. Powder Mater. **2**, 100091 (2023). <https://doi.org/10.1016/j.apmate.2022.100091>
[S13] Y. Li, R. Liu, X. Pang, X. Zhao, Y. Zhang et al., Fe@C nanocapsules with substitutional sulfur heteroatoms in graphitic shells for improving microwave absorption at gigahertz frequencies. Carbon **126**, 372-381 (2018). <https://doi.org/10.1016/j.carbon.2017.10.040>
